# Supplementary material for: Can Surgical Resection of Metastatic Lesions Be Beneficial to Pancreatic Ductal Adenocarcinoma Patients with Isolated Lung Metastasis?
Source: Cancers (Basel). 2022 Apr 20;14(9):2067. doi: 10.3390/cancers14092067 (PMC9099489; doi:10.3390/cancers14092067)
Supplement: Supplementary file 1 [file cancers-14-02067-s001.zip › cancers-1659974-supplementary.pdf]

Table S1. Demographics of PDAC patients with isolated pulmonary metastasis (Synchronous vs. Metachronous).

Variables	Synchronous(n=26)	Metachronous(n=57)	*P*-value
Age (Years) > 65	16 (61.5%)	27 (47.4%)	0.231
Sex (Male)	14 (53.8%)	25 (43.9%)	0.398
Tumor location (Head)	11 (42.3%)	31 (54.4%)	0.307
Tumor size (mm) > 28	12 (46.2%)	18 (31.6%)	0.200
Clinical N stage (cN+)	8 (30.8%)	8 (14.0%)	0.073
CEA (ng/ml) > 5	7 (26.9%)	12 (21.1%)	0.555
CA 19-9 (U/ml) > 37	24 (92.3%)	31 (24.4%)	0.001

Table S2. Demographics of PDAC patients with isolated pulmonary metastasis (Single vs. Oligometastases vs. Multiple).

Variables	Single(n=23)	Oligo(n=29)	Multiple(n=31)	*P*-value
Age (Years) > 65	13 (56.5%)	17 (58.6%)	13 (41.9%)	0.376
Sex (Male)	13 (56.5%)	9 (31.0%)	17 (54.8%)	0.102
Tumor location (Head)	11 (47.8%)	16 (55.2%)	15 (48.4%)	0.829
Tumor size (mm) > 28	6 (26.1%)	10 (34.5%)	14 (45.2%)	0.187
Clinical N stage (cN+)	4 (17.4%)	4 (13.8%)	8 (25.8%)	0.481
CEA (ng/ml) > 5	5 (21.7%)	8 (27.6%)	6 (19.4%)	0.741
CA 19-9 (U/ml) > 37	11 (47.8%)	22 (75.9%)	22 (71.0%)	0.082

(a)

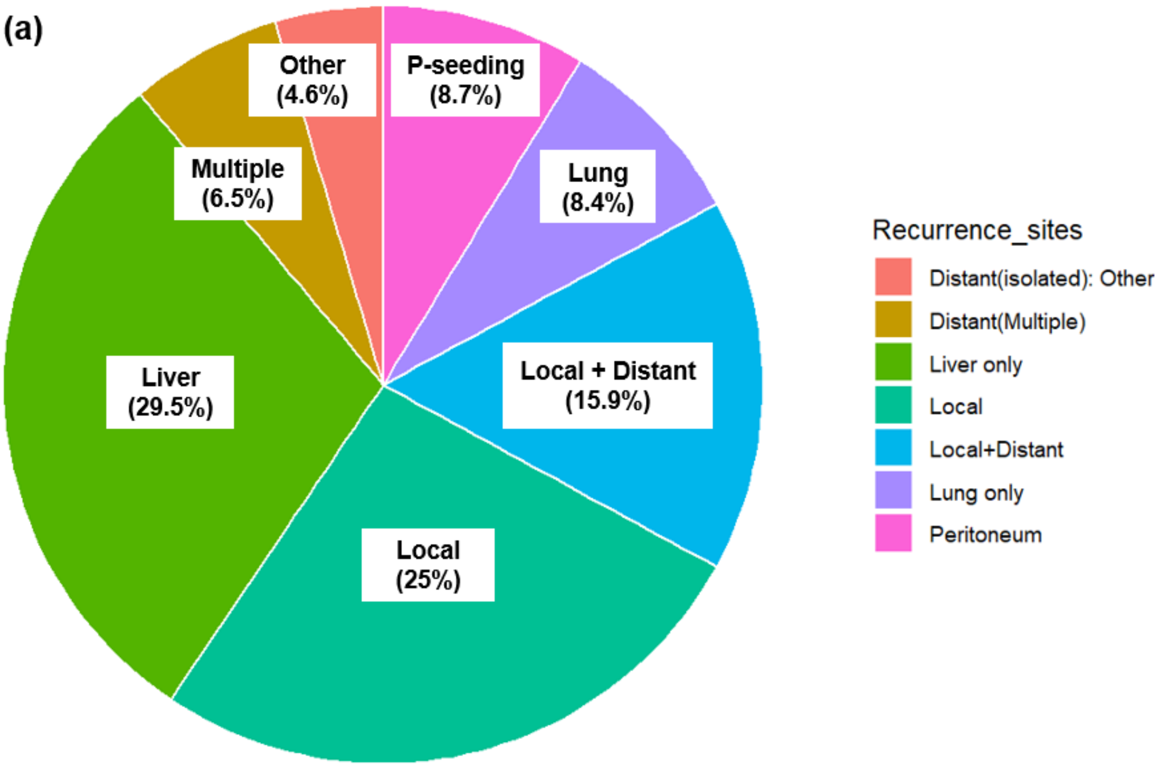

Other
(4.6%)
P-seeding
(8.7%)
Multiple
(6.5%)
Lung
(8.4%)
Liver
(29.5%)
Local + Distant
(15.9%)
Local
(25%)
Recurrence_sites
Distant(isolated): Other
Distant(Multiple)
Liver only
Local
Local+Distant
Lung only
Peritoneum

(b)

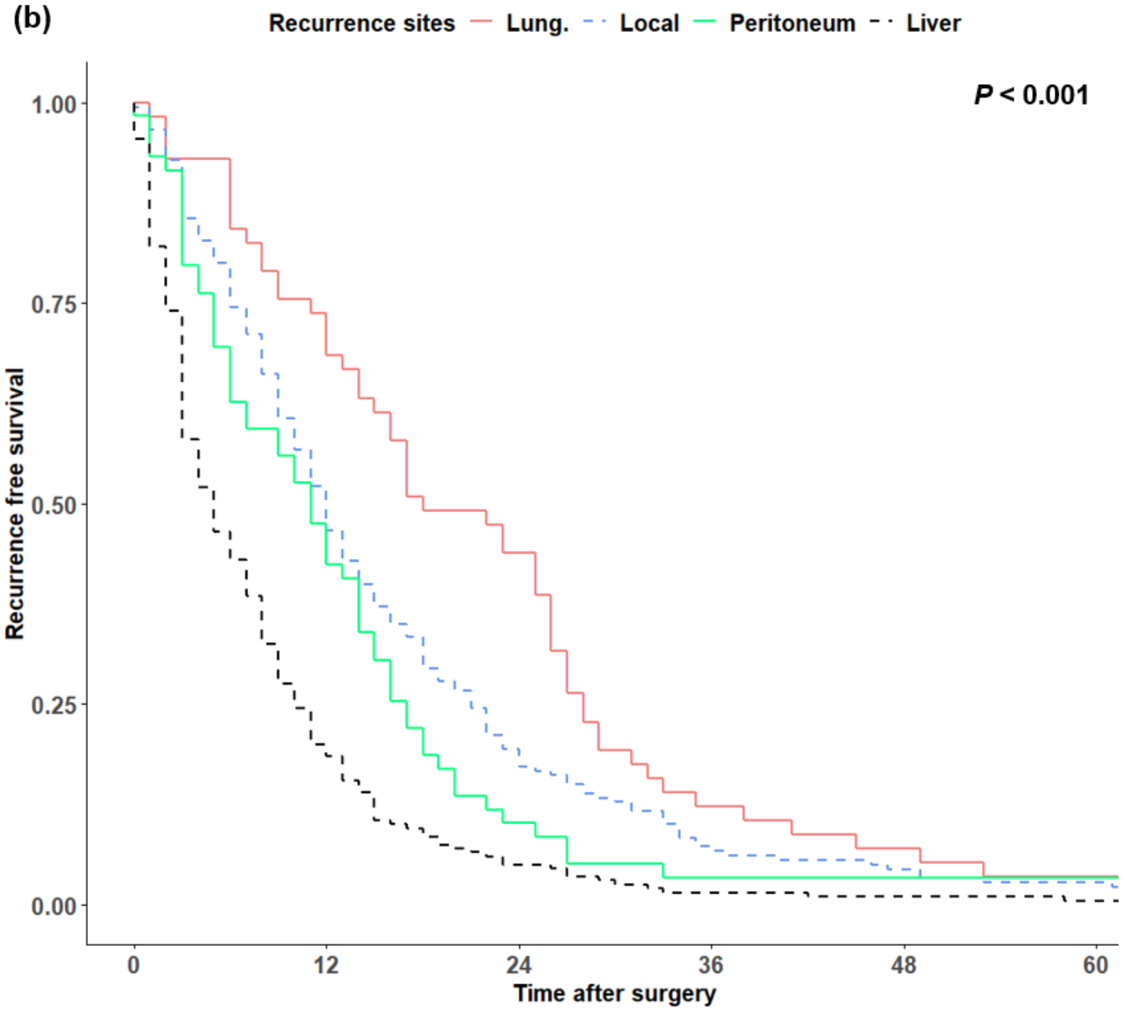

Recurrence sites
Lung.
Local
Peritoneum
Liver
P < 0.001
Recurrence free survival
1.00
0.75
0.50
0.25
0.00
0
12
24
36
48
60
Time after surgery

Figure S1. (**a**). Recurrence patterns after pancreatectomy in PDAC patients with metachronous metastasis. (**b**). Recurrence free survival in PDAC patients with metachronous metastasis confined to a specific site. Median recurrence free survival: Lung (18 months), Local (12 months), Peritoneal seeding (11 months), Liver (5 months).
